# Four Novel Leaderless Bacteriocins, Bacin A1, A2, A3, and A4 Exhibit Potent Antimicrobial and Antibiofilm Activities against Methicillin-Resistant Staphylococcus aureus

**DOI:** 10.1128/spectrum.00945-22

**Published:** 2022-08-24

**Authors:** Shu Liu, Shulin Deng, Hualin Liu, Liang Tang, Mengqi Wang, Bingyue Xin, Feng Li

**Affiliations:** a Anhui Province Key Laboratory of Pollutant Sensitive Materials and Environmental Remediation, College of Life Sciences, Huaibei Normal University, Huaibei, Anhui Province, China; b School of Marine Sciences, Sun Yat-sen University, Zhuhai, Guangdong Province, China; University of Pittsburgh

**Keywords:** methicillin-resistant *Staphylococcus aureus* (MRSA), leaderless bacteriocin, bacin

## Abstract

Methicillin-resistant Staphylococcus aureus (MRSA) is a major bacterial pathogen that causes hospital- and community-acquired infections. Owing to its multidrug resistance, it is imperative to develop new antimicrobial agents to treat MRSA infections. In this study, using genome mining analysis and a culture-based screening method to detect bacteriocin activity, we screened a strain, Bacillus sp. TL12, which harbored a putative leaderless bacteriocin gene cluster (*bac* gene cluster) and exhibited potent anti-MRSA activity. The antimicrobial agents, products of the *bac* gene cluster, were purified and identified as four novel leaderless bacteriocins: bacin A1, A2, A3, and A4. Bacin A2 was evaluated as a representative antimicrobial agent and showed remarkable antimicrobial activity against S. aureus, MRSA, and the foodborne pathogens Listeria monocytogenes and Bacillus cereus. Mechanistic experiments revealed that bacin A2 damaged cell membranes and exhibited bactericidal activity against MRSA. Bacin A2 effectively inhibited the formation of S. aureus and MRSA biofilms (>0.5× MIC) and killed the cells in their established biofilms (>4× MIC). The hemolytic and NIH/3T3 cytotoxicity assay results for bacin A2 confirmed its biosafety. Thus, bacins have potential as alternative antimicrobial agents for treating MRSA infections.

**IMPORTANCE** Methicillin-resistant Staphylococcus aureus (MRSA) is a major human pathogen that is difficult to treat because of its resistance to several widely used antibiotics. The present study aimed to identify novel anti-MRSA bacteriocins in a prominent producer of bacteriocins, Bacillus cereus group. Four novel leaderless bacteriocins, bacin A1, A2, A3, and A4, which show potent bactericidal effect against S. aureus and MRSA, were identified in Bacillus sp. TL12. Moreover, bacins inhibited biofilm formation and killed cells in the established biofilms of S. aureus and MRSA. These findings suggest that bacins are promising alternatives to treat MRSA infections.

## INTRODUCTION

Drug-resistant pathogens continue to be an important and growing threat to human health and economic development (1–3). The Centers for Disease Control and Prevention estimated that more than 70% of bacteria causing nosocomial infections are resistant to at least one of the drugs commonly used for treating these infections (4). According to a study chaired by Jim O’Neill, drug-resistant diseases cause more than 700,000 deaths/year (5). It is also estimated that by 2050, if there are no effective measures to delay the spread of drug resistance, 10 million people will die, and the economic loss will reach up to $100 trillion each year.

Staphylococcus aureus is a commensal bacteria of the skin and nasal flora in approximately 30% of healthy humans, and it is also an important opportunistic pathogen that causes skin and soft tissue infections, bacteremia, osteomyelitis, septic arthritis, pneumonia, endocarditis, and device-related infections (6–8). S. aureus is a highly adaptive and versatile pathogen with the ability to evolve resistance to a variety of antibiotics and form biofilms that protect against the host immune response and antimicrobial agents (9). A representative example is methicillin-resistant S. aureus (MRSA), which evolved from S. aureus after acquisition of the *mecA* gene from other microorganisms via horizontal gene transfer (8, 10, 11). Treatment of MRSA infections is challenging because of multidrug resistance to many commonly used antibiotics (virtually all β-lactam antibiotics and other classes of antibiotics, such as aminoglycosides, fluoroquinolones, and macrolides) and its protective biofilms (9, 12). Currently, MRSA is responsible for 10 times more infections than all multidrug-resistant Gram-negative pathogens combined (9). The World Health Organization indicated the mortality of MRSA-infected patients was 64% higher than that of drug-sensitive patients and listed MRSA as one of the 12 priority pathogens posing the greatest threat to human health. Thus, new anti-MRSA agents are urgently required (13).

Bacteriocins are ribosomally synthesized peptides produced by bacteria that show bacteriostatic or bactericidal activities against bacteria of the same species or other unrelated genera (14, 15). Bacteriocins have several properties that suggest they are promising antimicrobial agents in the treatment of infections caused by antibiotic-resistant pathogens. These properties include the availability of narrow- and broad-spectrum bacteriocins, their potent antimicrobial activities *in vitro* and *in vivo*, their low toxicity for the treated host, their distinct mode of actions that differ from those of antibiotics and thus are effective against both antibiotic-sensitive and -resistant bacteria, their stability over broad ranges of pH and temperature, and the feasibility of bioengineering of bacteriocins (14–17).

The Bacillus cereus group is comprised of a growing number of closely related species, including B. cereus, B. mycoides, B. pseudomycoides, B. thuringiensis, B. toyonensis, and B. weihenstephanensis (18, 19). Studies have shown that the sequenced genomes of the B. cereus group strains contain a diverse array of bacteriocins, and a variety of different kinds of bacteriocins have been identified and characterized (20–22). These bacteriocins generally show potent antimicrobial activity against Gram-positive strains, including some important foodborne (B. cereus, Listeria monocytogenes, etc.) and human pathogens (S. aureus, Enterococcus faecalis, Clostridium difficile, etc.) (20, 23–29). Although some bacteriocins have previously been investigated, research on the mining and identifying of novel bacteriocins with anti-MRSA activity is lacking, and the present study was designed to screen and identify novel bacteriocins with potent anti-MRSA activity produced by the B. cereus group. Eventually, four novel leaderless bacteriocins, bacin A1, A2, A3, and A4, with potent anti-MRSA activity were identified in Bacillus sp. TL12. The structure, biosafety, antimicrobial spectrum, and mode of action of bacins were measured, and the ability of these bacteriocins to prevent S. aureus and MRSA biofilm formation and kill the cells in their established biofilms were also investigated.

## RESULTS

### Screening of novel bacteriocin producers active against MRSA.

**FIG 1:**
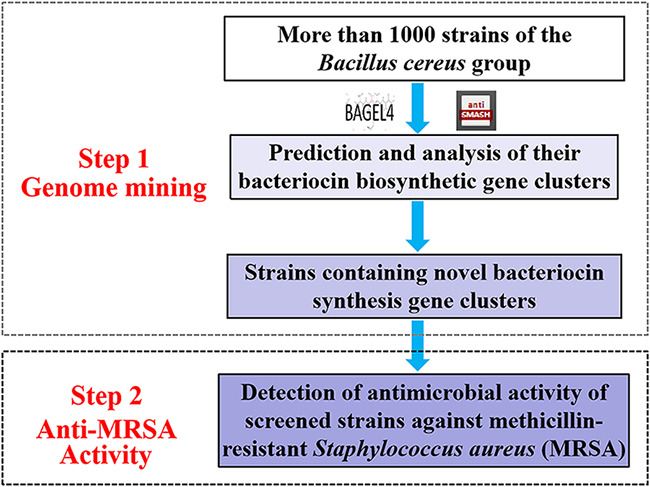
Screening strategy for novel bacteriocin-producing strains of the Bacillus cereus group with antimicrobial activity against methicillin-resistant Staphylococcus aureus (MRSA).

A two-step approach was used to screen the B. cereus group strains that produce novel bacteriocins with anti-MRSA activity (Fig. 1). In step 1, 1,056 strains of the B. cereus group underwent genome mining to predict and analyze their bacteriocin synthesis gene clusters. In step 2, the strains containing novel bacteriocin gene clusters were cultured in three liquid media (Luria-Bertani broth [LB], nutrient broth [NB], and trypticase soy broth [TSB]), and the antimicrobial activity of their supernatants against the MRSA standard strain ATCC 43300 was tested. Of these, 502 strains were predicted to contain novel bacteriocin synthesis gene clusters, and the types of bacteriocins included lantipeptides, lasso peptides, sactibiotics, linear azol(in)e-containing peptides, thiopeptides, glycocins, circular bacteriocins, leaderless bacteriocins, and class III bacteriocins (data not shown). Further anti-MRSA activity testing showed that the supernatants of 56 strains exhibited antimicrobial activity against MRSA (Table S1 in supplemental material).

**FIG 2:**
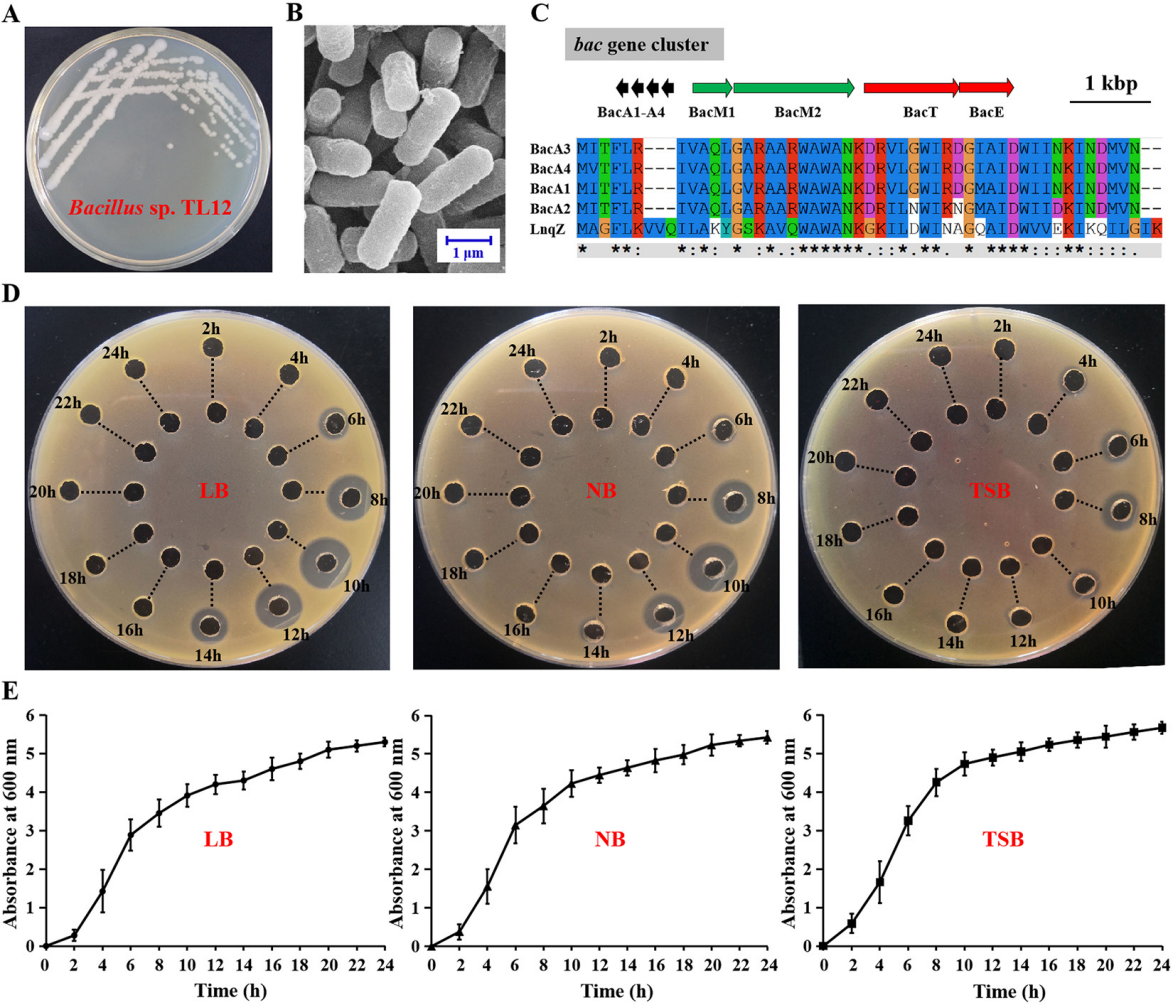
Screening of the bacteriocin-producing strain Bacillus sp. TL12. (A) Colony morphology of Bacillus sp. TL12. (B) Scanning electron microscope observation of Bacillus sp. TL12. (C) The putative leaderless bacteriocin gene cluster (*bac* gene cluster) in the genome of Bacillus sp. TL12 and multiple-sequence alignments of their precursor peptides (BacA1, BacA2, BacA3, and BacA4) with LnqZ (the precursor peptide of a leaderless bacteriocin, Lacticin Z). Black, precursor peptides; red, transport or immune related proteins; green, membrane proteins. (D) Antimicrobial activity of the supernatant of Bacillus sp. TL12 against MRSA ATCC 43300. The outer well was added to the untreated supernatant, and the inner well was added to the corresponding supernatant treated with mixed enzymes. (E) Growth kinetics of Bacillus sp. TL12 in Luria-Bertani broth (LB), nutrient broth (NB), and trypticase soy broth (TSB) media. All experiments were performed in triplicate, and the data are shown as the means ± SD.

Among these 56 strains, one, Bacillus sp. TL12, contained a putative leaderless bacteriocin gene cluster (termed the *bac* gene cluster) and showed the highest anti-MRSA activity (Fig. 2A to E). The *bac* gene cluster encoded four highly homologous precursor peptides, BacA1, BacA2, BacA3, and BacA4 (amino acid sequence identities ranged from 85.42 to 97.92%), which exhibited 45 to 47% identities to LnqZ, the precursor peptide of leaderless bacteriocin, lacticin Z (30) (Fig. 2C). TL12 produced anti-MRSA substances in all three tested liquid media (LB, NB, and TSB) during the exponential growth phase, and the antimicrobial substances could be degraded by mixed proteases, indicating their peptide or protein properties (Fig. 2D and E). In comparison, among the three tested media, the TL12 strain could produce the largest amount of antimicrobial substances (maximum antimicrobial production was recorded at 10 h), and the detectable antimicrobial activity lasted the longest time (activity was detected between 6 and 14 h) when cultured in LB medium (Fig. 2D). In conclusion, Bacillus sp. 12 was regarded as a novel bacteriocin-producing strain with anti-MRSA activity and was used for subsequent analysis.

### Bacillus sp. TL12 produced four novel leaderless bacteriocins: bacin A1, A2, A3, and A4.

**FIG 3:**
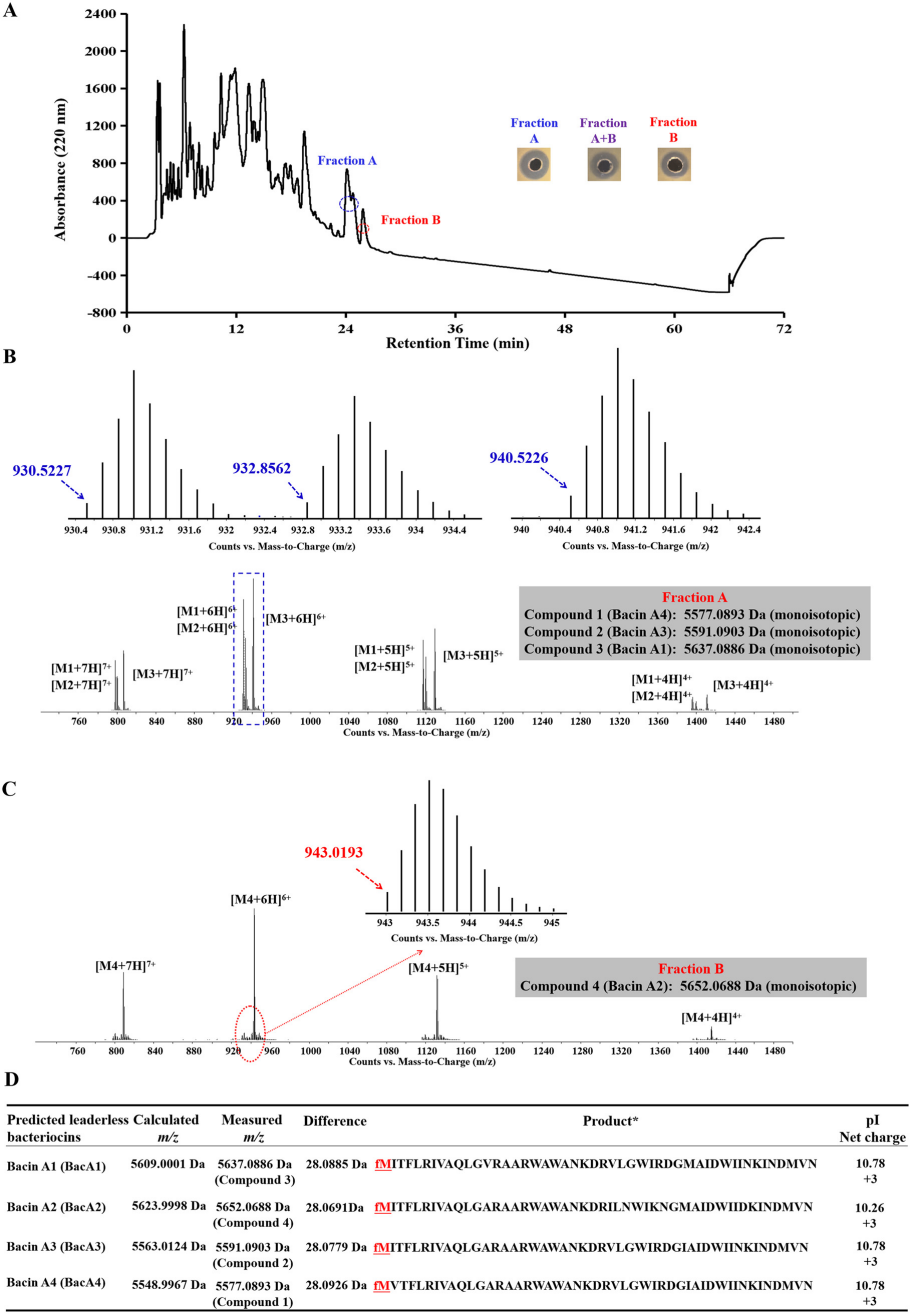
Purification and identification of four novel leaderless bacteriocins produced by Bacillus sp. TL12. (A) High-performance liquid chromatography (HPLC) analysis of a CE of Bacillus sp. T1L12 culture supernatant. Fractions A and B have antimicrobial activity against MRSA ATCC 43300. No synergistic antimicrobial effect was observed between fractions A and B. (B) Mass spectrometry (MS) analysis of antimicrobials in fraction A. (C) MS analysis of antimicrobials in fraction B. (D) Analysis of the molecular mass of four putative leaderless bacteriocins of Bacillus sp. TL12. fM, formylmethionine.

The antimicrobial substance in the culture supernatant (grown in LB medium for 10 h) of Bacillus sp. TL12 was concentrated with Amberlite XAD-7 HP resin, and the acquired antimicrobial crude extract (CE) was separated by reverse-phase high-performance liquid chromatography (RP-HPLC). As shown in Fig. 3A, two fractions (fractions A and B) showed antimicrobial activity against MRSA 43300, and no synergistic antimicrobial effect was observed between fractions A and B. Liquid chromatography/mass spectrometry (LC/MS) analysis revealed that fraction A consisted of three compounds with monoisotopic masses of 5,577.0893 Da (compound 1), 5,591.0903 Da (compound 2), and 5,637.0886 Da (compound 3), respectively (Fig. 3B). Fraction B consisted of a pure compound with a monoisotopic mass of 5,652.0688 Da (compound 4) (Fig. 3C). The calculated molecular masses of four predicted mature peptides of BacA1, BacA2, BacA3, and BacA4 (termed bacin A1, A2, A3, and A4, respectively) were 5,609.0001, 5,623.9998, 5,563.0124, and 5,548.9967 Da, which were approximately 28 Da smaller than that of the four antimicrobials (compounds 3, 4, 2, and 1, respectively) of TL12, suggesting that the antimicrobial substances produced by TL12 are four leaderless bacteriocins, and the N-terminal of these bacteriocins underwent formylation modification (Fig. 3D).

**FIG 4:**
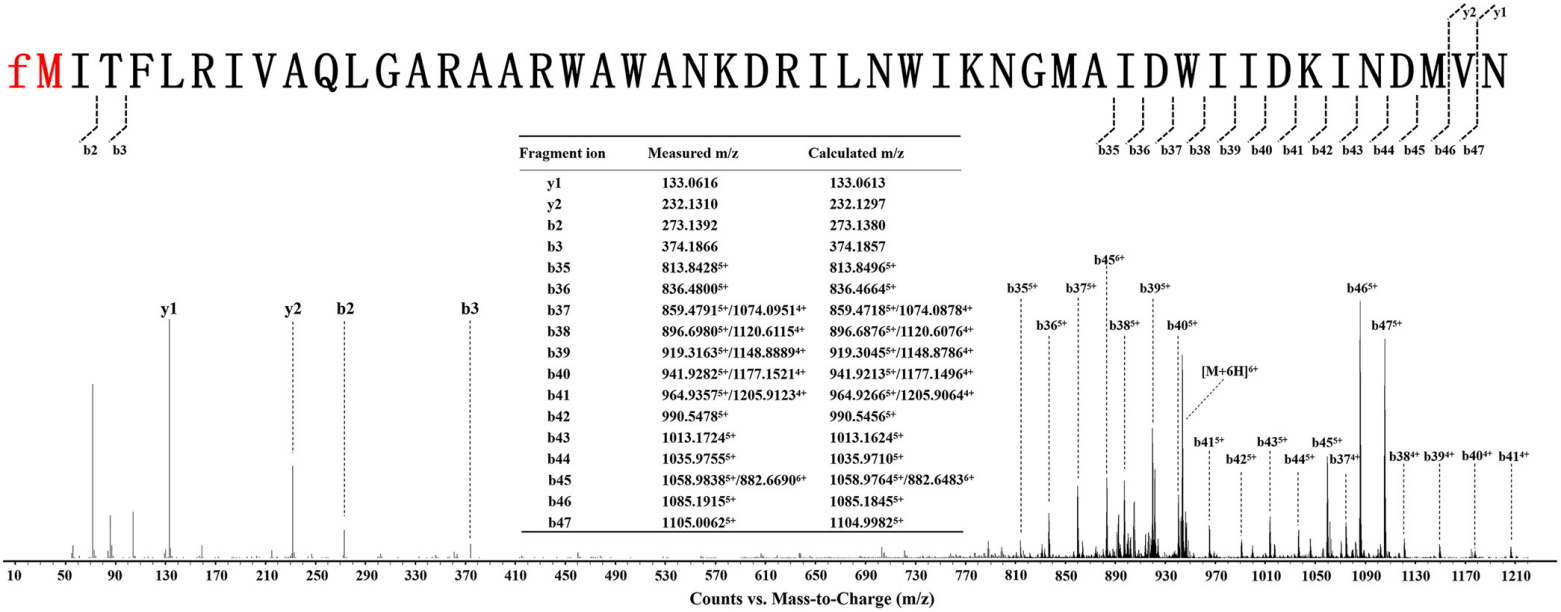
MS/MS spectra and proposed structure of bacin A2. The *b* and *y* ions in the MS/MS spectra of bacin A2 were manually assigned upon fragmentation.

To further verify the speculation on the structure of four antimicrobial substances, we took compound 4 as a representative and performed amino acid sequencing and tandem mass spectrometry (MS/MS) analysis. No amino acid residues were obtained by N-terminal amino acid sequencing (Edman degradation) of compound 4 (data not shown). MS/MS analysis showed that the molecular masses of a series of fragment ions of compound 4 (b2, b3, b35, b36, b37, b38, b39, b40, b41, b42, b43, b44, b45, b46, and b47) were approximately 28 Da higher than those of the corresponding fragment ions of BacA2 (Fig. 4). To sum up the above-described experimental results, we concluded that the antimicrobial substances produced by TL12 is the product of *bac* gene cluster—the four novel leaderless bacteriocins bacin A1, A2, A3, and A4 and their N-terminal amino acid is formylmethionine (fM).

### Antimicrobial activity of bacin A2.

Bacin A1, A3, and A4 showed high structural similarity and existed in the same chromatographic peak in HPLC analysis, and only pure bacin A2 could be obtained using HPLC analysis (Fig. 3A). Therefore, the high purity of bacin A2 achieved by repeated HPLC collection and preparation was used as the test and analysis object in subsequent experiments (Fig. S1). The antimicrobial activity of bacin A2 for various Gram-positive and Gram-negative strains was tested using the microdilution method. Bacin A2 displayed potent antimicrobial activity against multiple Gram-positive strains, including S. aureus ATCC 6538 (MIC, 0.5 μM; MBC, 1 μM), S. aureus ATCC 12600 (MIC, 1 μM; MBC, 2 μM), S. aureus ATCC 43300 (MRSA) (MIC, 1 μM; MBC, 2 μM), S. epidermidis CMCC 26069 (MIC, 1 μM; MBC, 2 μM), B. cereus ATCC 14579 (MIC, 0.5 μM; MBC, 2 μM), B. cereus CMCC 63303 (MIC, 0.5 μM; MBC, 2 μM), L. monocytogenes ATCC 19111 (MIC, 0.5 μM; MBC, 2 μM), and L. monocytogenes ATCC 19115 (MIC, 0.5 μM; MBC, 2 μM); exhibited modest antimicrobial activity against Streptococcus pyogenes ATCC 19615 (MIC, 8 μM; MBC, 32 μM), E. faecalis ATCC 29212 (MIC, 16 μM; MBC, 32 μM), and E. faecalis ATCC 51299 (VRE) (MIC, 16 μM; MBC, 32 μM); and had no antimicrobial effect on all tested Gram-negative strains even at the high concentration of 128 μM (Table 1).

**TABLE 1:** MICs and MBCs of bacin A2

Indicator stains and genotype^*a*^	Medium	Bacin A2 MIC (μM)^*b*^	Bacin A2 MBC (μM)^*b*^
Bacillus cereus ATCC 14579	LB	0.5	2
Bacillus cereus CMCC 63303	LB	0.5	2
Listeria monocytogenes ATCC 19115	TSB-YE	0.5	2
Listeria monocytogenes ATCC 19111	TSB-YE	0.5	2
Staphylococcus aureus ATCC 6538	NB	0.5	1
Staphylococcus aureus ATCC 43300 (MRSA)	NB	1	2
Staphylococcus aureus ATCC 12600	NB	1	2
Staphylococcus epidermidis CMCC 26069	NB	1	2
Streptococcus pyogenes ATCC 19615	BHI	8	32
Enterococcus faecalis ATCC 29212	BHI	16	32
Enterococcus faecalis ATCC 51299 (VRE)	BHI	16	32
Acinetobacter baumannii ATCC 19606	NB	NA	NA
Escherichia coli ATCC 25922	NB	NA	NA
Pseudomonas aeruginosa ATCC 27853	NB	NA	NA
Salmonella enterica serotype Paratyphi CMCC 50093	NB	NA	NA
Salmonella Typhimurium ATCC 14028	NB	NA	NA
Serratia marcescens CMCC 41002	NB	NA	NA
Shigella serogroups CMCC 51105	NB	NA	NA

a ATCC, American Type Culture Collection; CMCC, China Medical Culture Collection; MRSA, methicillin-resistant S. aureus; VRE, vancomycin-resistant Enterococci; LB, Luria-Bertani broth; NB, nutrient broth; BHI, brain-heart infusion broth; TSB-YE, tryptic soy broth with yeast extract.

b NA, no antimicrobial activity was observed even at the highest concentration of 128 μM.

### Bacin A2 exerts bactericidal activity by damaging the membrane integrity.

**FIG 5:**
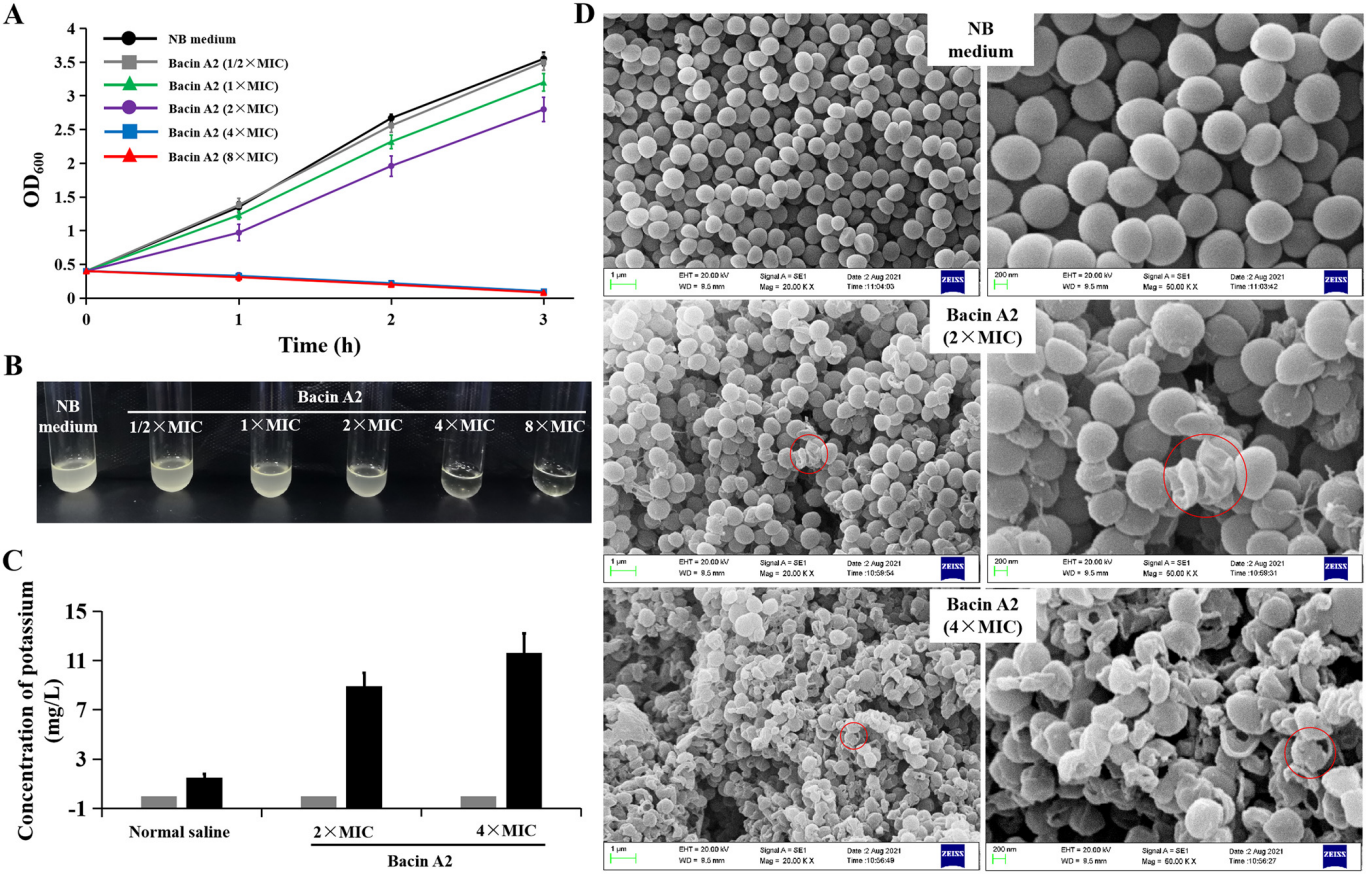
Bacin A2 targets the cell membrane of MRSA cells and exerts bactericidal activity. (A) Growth curve of MRSA ATCC 43300 treated with various concentrations of bacin A2. Bacin A2 was dissolved and diluted with NB medium. NB medium was used as the negative control. The data are expressed as the means ± SD of three independent replicates. (B) Bacin A2 treatment resulted in the lysis of MRSA cells. The figure is representative of three independent experiments. (C) Potassium release from MRSA cells after treated with 2× and 4× MIC of bacin A2. Bacin A2 was dissolved and diluted with normal saline. Normal saline was used as the negative control. The data are expressed as the means ± SD of three independent replicates. (D) Morphology observation of MRSA ATCC 43300 cells after treated with 2× and 4× MIC of bacin A2 with SEM. Bacin A2 was dissolved and diluted with NB medium. NB medium was used as the negative control. The red circles indicate the significant membrane damage of MRSA cells.

To investigate the mode of action of bacin A2, the cell growth, potassium ion release, and cell morphological change of MRSA ATCC 43300 cells when incubated under different concentrations of bacin A2 were measured and analyzed. As shown in Fig. 5A and B, the growth of MRSA ATCC 43300 was inhibited by bacin A2 in a dose-dependent manner; the growth of MRSA ATCC 43300 was not affected when the concentration of bacin A2 was 1/2× MIC; the growth of MRSA ATCC 43300 was significantly inhibited when the concentration of bacin A2 was 1× MIC to 2× MIC; the growth of MRSA ATCC 43300 was completely inhibited when the concentration of bacin A2 was 4× MIC or 8× MIC; and the culture gradually cleared with the increase in incubation time. These results indicate that bacin A2 showed a bactericidal effect against MRSA ATCC 43300 cells. In addition, bacin A2 can cause significant membrane damage of MRSA ATCC 43300 cells. The amount of released potassium ion was significantly increased when S. aureus cells were treated with bacin A2 for 3 h (control, 1.5 mg/mL; 2× MIC, 8.9 mg/mL; 4× MIC, 11.6 mg/mL) (Fig. 5C). Scanning electron microscopy (SEM) analysis demonstrated that the cell membrane of MRSA ATCC 43300 cells became deformed, collapsed, and even lysed when incubated with bacin A2 (2× MIC and 4× MIC) for 3 h, whereas the untreated control cells showed smooth and intact surfaces (Fig. 5D). In conclusion, bacin A2 causes cell membrane damage and exerts bactericidal activity on MRSA strains.

### Effect of bacin A2 on S. aureus biofilm.

**FIG 6:**
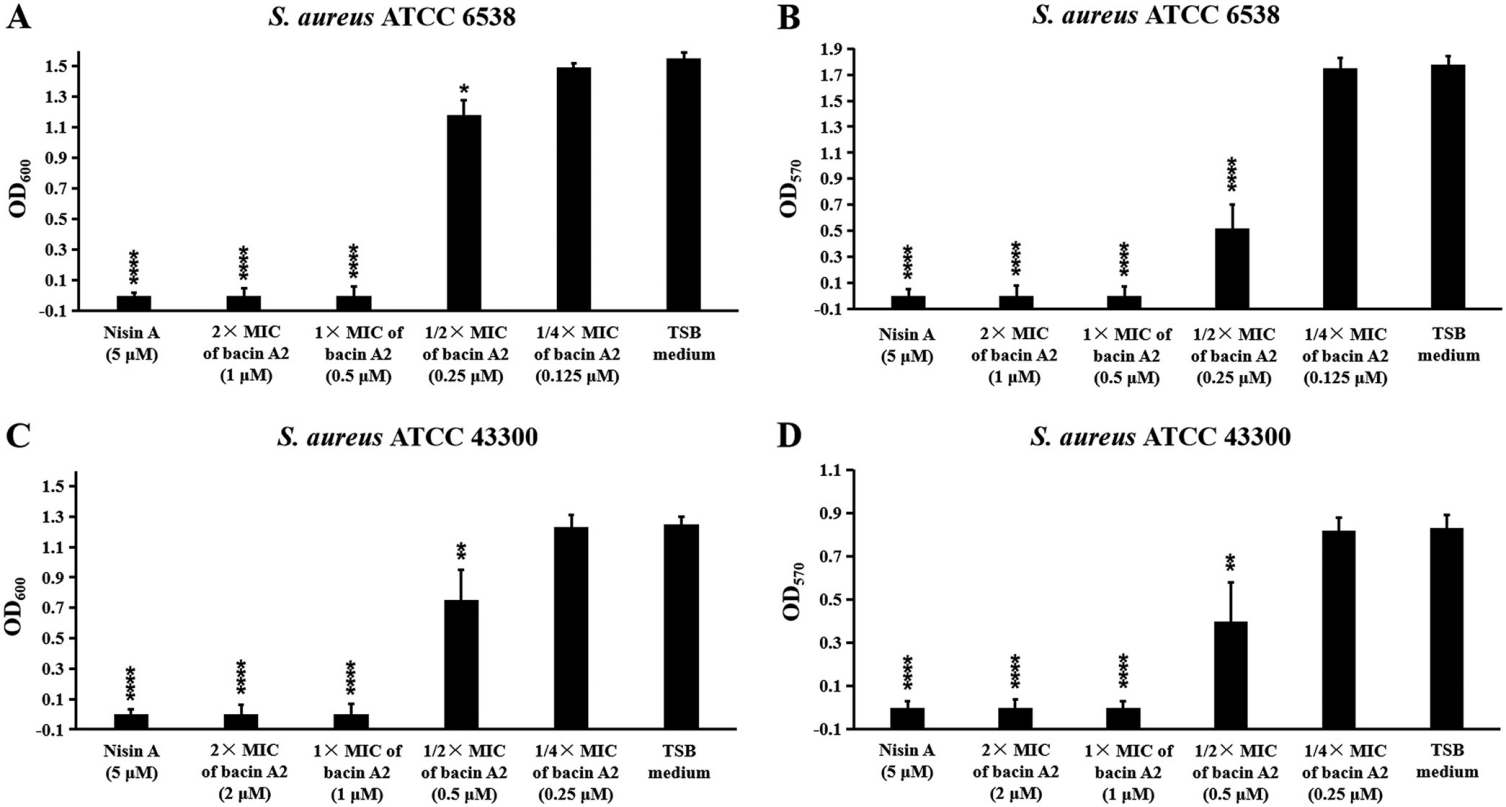
Growth and biofilm formation of S. aureus ATCC 6538 (A, B) and MRSA ATCC 43300 (C, D) in the presence of bacin A2 (1/4×, 1/2×, 1×, 2× MIC). Bacin A2 was dissolved and diluted with TSB medium. Nisin A (5 μM) and TSB medium were used as positive and negative controls, respectively. Asterisk ratings indicate statistically significant differences: *, *P* ≤ 0.05; **, *P* ≤ 0.01; ***, *P* ≤ 0.001; and ****, *P* ≤ 0.0001.

The effects of bacin A2 on biofilm formation of S. aureus ATCC 6538 and MRSA ATCC 43300 were examined by crystal violet (CV) assay. As shown in Fig. 6, at 1/4× MIC of bacin A2, the growth and biofilm formation of S. aureus ATCC 6538 and MRSA ATCC 43300 were not affected (*P > *0.05); at 1/2× MIC of bacin A2, the growth and biofilm formation of S. aureus ATCC 6538 and MRSA ATCC 43300 were all significantly inhibited compared to the untreated control (*P < *0.05); and at 1× MIC and 2× MIC of bacin A2 or 5 μM Nisin A (positive control), the growth of S. aureus ATCC 6538 and MRSA ATCC 43300 was completely prevented with no biofilm formation (*P < *0.0001). These results demonstrated that bacin A2 could effectively inhibit the biofilm formation of S. aureus and MRSA.

**FIG 7:**
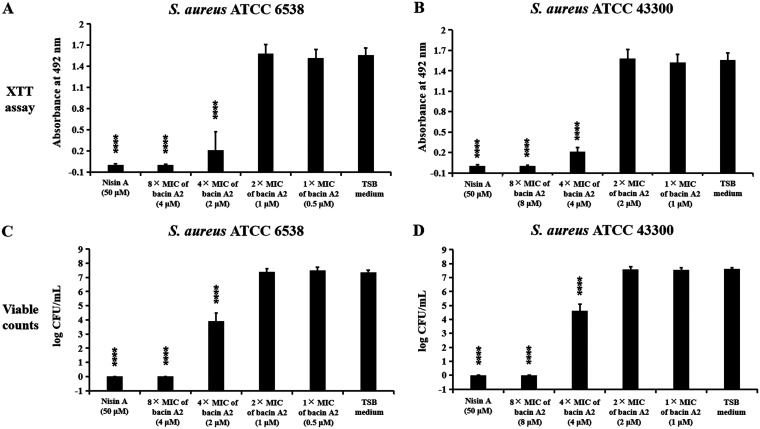
The effect of bacin A2 on cell viability in 1-day-old biofilms of S. aureus ATCC 6538 and MRSA 43300. The viability of bacterial cells in 1-day-old biofilms was detected by 2,3-bis-(2-methoxy-4-nitro-5-sulfophenyl)-2H-tetrazolium-5-carboxanilide salt (XTT) assay (A, B) and viable count (C, D). Bacin A2 was dissolved and diluted with TSB medium. Nisin A (50 μM) and TSB medium were used as positive and negative controls, respectively. ****, *P* ≤ 0.0001.

The effects of bacin A2 on 1-day-old preformed biofilms of S. aureus ATCC 6538 and MRSA ATCC 43300 were examined by the direct method (viable counting) and indirect method (the 2,3-bis-(2-methoxy-4-nitro-5-sulfophenyl)-2H-tetrazolium-5-carboxanilide salt [XTT] assay). As shown in Fig. 7A and B, significantly reduced XTT conversion was observed with 4× MIC of bacin A2 (*P < *0.0001), and no XTT conversion was observed under 8× MIC of bacin A2 or 50 μM Nisin A (positive control) (*P < *0.0001). The results of the viable count method correlated with the results of XTT assay, and the number of live S. aureus cells in biofilms was decreased by more than 3-log at 4× MIC of bacin A2; no viable cells were detected at a high concentration of bacin A2 (8× MIC) or 50 μM Nisin A (positive control) (Fig. 7C and D). These results demonstrated that bacin A2 could effectively kill S. aureus and MRSA cells in preformed biofilms.

### Hemolytic activity and cytotoxicity of bacin A2.

**FIG 8:**
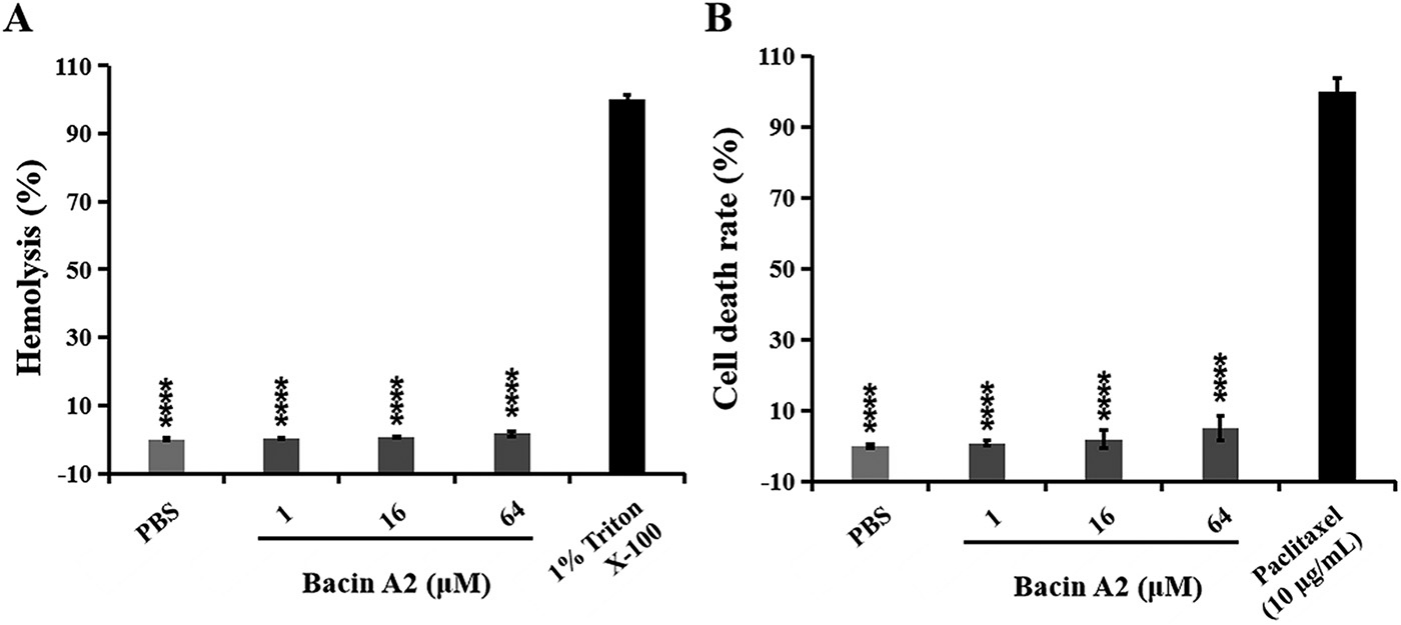
Hemolytic activity and cytotoxicity of bacin A2. Hemolytic activity of bacin A2 in sheep blood cells (A). Bacin A2 was dissolved and diluted with phosphate-buffered saline (PBS). PBS was taken as the negative control, and 1% Triton X-100 was taken as the positive control. Cytotoxicity of bacin A2 in NIH/3T3 cells (B). Bacin A2 was dissolved and diluted with PBS. PBS was taken as the negative control, and paclitaxel (10 μg/mL) was taken as the positive control. ****, *P ≤ *0.0001.

The hemolytic activity of bacin A2 was measured using sheep red blood cells. The hemolysis rates of bacin A2 at 1, 16, and 64 μM were 0.3, 0.5, and 1.63%, respectively, all significantly different from that of the positive control, 1% Triton X-100 (*P < *0.0001) (Fig. 8A). The cytotoxicity of bacin A2 was analyzed using CCK-8 assay on NIH/3T3 cells. The cell death rates of bacin A2 at 1, 16, and 64 μM in NIH/3T3 cells were 0.7, 1.8, and 4.9%, respectively, all significantly different from that of paclitaxel (positive control) (*P < *0.0001) (Fig. 8B).

## DISCUSSION

MRSA is a global public health problem that has urged researchers to discovery new and effective antimicrobial agents to combat it (6–13). In this study, we used genome mining analysis and a culture-based screening method for detecting bacteriocin activity to screen the B. cereus group strains that produce novel bacteriocins with anti-MRSA activity and further identify these novel bacteriocins. Considering that a dozen bacteriocins have been reported in the B. cereus group strains, this approach could exclude the strains expressing only previously reported bacteriocin synthetic gene clusters to reduce the workload and improve screening efficiency (29). In addition, we noticed that only 56 of the 502 strains containing novel bacteriocin gene clusters showed noticeable anti-MRSA activity. We consider that there are two reasons for this phenomenon. First, although some strains produce a large amount of bacteriocin, these bacteriocins have no activity against MRSA. Second, the bacteriocin gene cluster in some bacteria is not expressed, or the expression is insufficient to monitor the activity against MRSA under the culture conditions used in this study. This situation has also been reported in other studies and requires optimization of the culture conditions (such as culture medium, temperature, and pH) or a semi-*in vitro* biosynthesis strategy to obtain bacteriocin products (24, 26, 31). Among the 56 strains with anti-MRSA activity, strain TL12 had the highest anti-MRSA activity and was therefore used in this study. The other 55 strains contain the putative bacteriocin gene clusters, which code for lantipeptides, lasso peptides, linear azol(in)e-containing peptides, thiopeptides, circular bacteriocins, and leaderless bacteriocins (data not shown), and we will continue to identify these novel bacteriocins in future studies.

Through MS and MS/MS analysis, we determined that the N-terminal of bacins was formylated methionine. Notably, the N termini of most reported leaderless bacteriocins were formylated methionine. Some studies have revealed that the formylation of methionine at the N-terminal of leaderless bacteriocins may not play a significant role in their antimicrobial activities (32, 33). For example, aureocin A53 and lacticin Q with non-fM at their N termini obtained by a Escherichia coli expression system still have intact antimicrobial activities. We will use E. coli and/or a yeast expression system to produce bacins with non-fM at their N termini and compare their antimicrobial activities in further research. In addition, there are no genes coding for formylase in all of the reported synthetic gene clusters of N-terminally formylated leaderless bacteriocins, including the bacin gene cluster in this study (33). This suggests that the enzyme responsible for formylation modification is the host-encoded formylase, meaning the gene is located outside the bacteriocin gene cluster.

Bacin A1, A2, A3, and A4 have high amino acid identity; bacin A1, A3, and A4 show particularly high homology, with differences within only three amino acids. Indeed, these three are so close that their respective pure products cannot be separated by HPLC (Fig. 3A). Currently, we can obtain only the pure product of bacin A2, and therefore, bacin A2 was used as representative for all the activity test in this study. Bacin A2 exhibits potent antimicrobial activity against S. aureus and MRSA (Table 1). S. aureus is a major human pathogen that causes a variety of infections, and the emergence of drug-resistant strains of S. aureus, especially MRSA, makes this organism a dangerous pathogen that can prove fatal due to lack of alternative antibiotics (6–13). The potent anti-MRSA activity of bacins indicates that bacins are promising antimicrobial agents for the treatment of infection of MRSA. In addition, bacins also show potent antimicrobial activity against two foodborne pathogens: B. cereus and L. monocytogenes. B. cereus can produce emetic toxin (cereulide) and diarrheal enterotoxins and cause a self-limiting gastrointestinal disorder (vomiting and diarrhea) (34, 35). L. monocytogenes is a leading foodborne pathogen that produces a series of virulence factors (Listeria adhesion protein, internalin, phospholipase, and actin polymerization protein) and causes gastroenteritis, systemic listeriosis, abortion in pregnant women, and neonatal listeriosis is fetuses (36). In addition, S. aureus is also an important foodborne pathogen that can produce a large number of toxins (enterotoxins, exfoliative toxins, and pore-forming hemolysins) and enzymes (collagenase, endopeptidase, and metalloprotease) and cause severe cramping and vomiting with or without diarrhea (37–39). Therefore, bacins not only are considered a promising anti-MRSA drug but also have potential as a natural food preservative in food production.

The mode of action of bacin A2 was studied using a standard MRSA strain, S. aureus ATCC 43300, as the indicator strain. The growth and time-kill curve assays revealed that bacin A2 is a bactericidal substance. SEM analysis revealed that the cell morphology becomes irregular and even cleaved when treated with 2× MIC or 4× MIC of bacin A2. Moreover, efflux of intracellular potassium ions could be detected after the treatment of bacin A2. Combining the high isoelectric points (pI 10.26) and net positive charge (+3) at physiological pH of bacin A2 (Fig. 3D), we deduced its mode action: first, the negatively charged cell membrane is contacted electrostatically; then, pores are formed, leading to the leakage of solutes and ions and eventually cell death.

Biofilms are formed from a community of microorganisms embedded in a self-produced extracellular polymeric substance (including polysaccharides, extracellular DNA, proteins, and lipids) that adhere to living or nonliving surfaces (40). S. aureus can form biofilms on medical devices and host tissue, and its biofilms act as a physical barrier and provide protection from antibiotics and the host immune system (41, 42). Therefore, activity on S. aureus biofilms is an important characteristic when developing new drugs. Strategies to combat S. aureus biofilms were divided into two categories: prevention of biofilm formation and elimination of established S. aureus biofilms (9), and we paid close attention to the activities of bacins against biofilms and biofilm formation of S. aureus and MRSA strains. Our results confirmed that bacin A2 at 1/2× MIC significantly reduced biofilm formation, and bacin A2 at 1× MIC and 2× MIC completely inhibited biofilm formation. The biomass of the corresponding experimental group also decreased, indicating that bacin A2 inhibited biofilm formation by inhibiting bacterial growth. This phenomenon was also observed in several other reported antimicrobial agents (43–45). When applied on established S. aureus biofilms, bacin A2 at 4× MIC was able to significantly reduce biofilm cell viability, and bacin A2 at 8× MIC was able to completely kill the cells in the biofilms. The potent activity on S. aureus and MRSA biofilms indicated that bacins have the potential to prevent or treat biofilm-associated infections.

Bacteriocins are important virulence factors in some bacterial pathogens and show antimicrobial activity along with hemolysis and/or cytotoxicity. Examples include BacSp222 of Staphylococcus pseudintermedius (46), cytolysin of E. faecalis (47), and streptolysin S of group A Streptococcus (48). Therefore, determining the biosafety of bacteriocin is vital for its development and application in medicine or food. *In vitro* hemolysis and cell viability assays showed that bacin A2 had negligible hemolytic activity and cytotoxicity at the concentration at which it exerted anti-MRSA activity (1 μM). Furthermore, it showed low hemolytic activity and cytotoxicity even at high concentrations (16 and 64 μM). This demonstrated that bacins are antimicrobial substances with biosafety, which can be further explored for practical application in health care and industry.

## MATERIALS AND METHODS

### Screening of anti-MRSA bacteriocin-producing strains of the B. cereus group.

We screened a total of 1,056 strains of the B. cereus group for bacteriocin production, which were isolated from various types of soil samples, including forest, riverside, farmland, and wetland from different regions of China by a classical heat treatment method and preserved in our laboratory (Table S1 in supplemental material). The genome of these strains was sequenced by Wuhan Grandomics Biological Technology Co. using the Illumina HiSeq X 10 platform, and sequence reads were assembled through PGCGAP 1.0.34 (https://liaochenlanruo.fun/pgcgap/index-v1.0.34.html). Species identification of these strains was performed using a 16S rRNA gene sequence and comparative genome analyses (average nucleotide identity and digital DNA-DNA hybridization [DDH] analyses between genome sequences of the strains and type strain of a species). The species of these strains included B. albus, B. bombysepticus, B. cereus, B. mycoides, B. thuringiensis, B. toyonensis, B. pseudomycoides, B. weihenstephanensis, and some other unidentified species (Table S1).

The screening strategy for novel bacteriocin-producing strains with anti-MRSA activity was divided into two steps (Fig. 1). In step 1, the genomes of 1,056 strains were uploaded to the online software antiSMASH 5.0 (49) and BAGEL4 (50) to identify the strains containing novel bacteriocin synthesis gene clusters, which are needed to satisfy two conditions. First, the gene cluster must contain necessary bacteriocin synthesis related genes, such as structural genes, modification, transport, and immune related genes. The function of each gene in the bacteriocin synthesis gene cluster was analyzed using BLAST software. Second, the novelty of bacteriocin synthesis gene cluster must be through analyzing the amino acid sequence identity between the precursor peptide encoded by the gene cluster and the reported bacteriocin precursor peptide; the precursor peptide of bacteriocin had less than 60% identity with the reported bacteriocin precursor peptide. In step 2, using MRSA ATCC 43300 as the indicator strain, the antimicrobial activity of the above screened strains was analyzed. Briefly, the screened strains were activated overnight in LB, TSB, and NB media, and the culture (1 mL) from each medium was transferred into LB, TSB, and NB media (100 mL), respectively, and incubated for 24 h (30°C) with shaking at 200 rpm. Cell growth was monitored every 2 h by measuring the absorbance of fermentation broth at 600 nm. The antimicrobial activity of the supernatants (2 to 24 h) against MRSA was measured by the agar well diffusion method (21). Briefly, 20 mL LB agar (45°C) containing 1.0 × 10^6^ cells was spread onto plates. The wells were punched and filled up with the supernatants. The plates were incubated at 4°C for 2 h and then at 30°C for 16 h. The diameter of the inhibition zone was measured to assess the antimicrobial activity of supernatant during the growth. The supernatants with antimicrobial activities were then incubated with mixed proteases (trypsin and α-chymotrypsin at concentrations of 1 mg/mL) at 37°C for 3 h. Residual antimicrobial activity was measured using the agar well diffusion method as described earlier.

### Anti-MRSA bacteriocin-producing strain Bacillus sp. TL12 and the *bac* gene cluster.

One strain, TL12, which was isolated from the soil of Xiangshan Mountain, Huaibei City, Anhui Province, China, contained a putative leaderless bacteriocin gene cluster (termed the *bac* gene cluster) and could produce anti-MRSA substances that could be degraded by mixed proteases (Fig. 2). The 16S rRNA gene sequences of TL12 (GenBank accession no. OL774512.1) had high homology with different strains of the B. cereus group, such as B. anthracis BJHS4 (99.9%), B. cereus D2-5 (99.9%), B. mycoides strain S20704 (99.9%), and B. thuringiensis FDAARGOS_792 (99.8%). It was impossible to confirm the species with just 16S rRNA gene sequence analysis. The average nucleotide identity and digital DNA-DNA hybridization between genome sequences of the TL12 strain and the most closely related strain, B. pseudomycoides BTZ, were 89.72 and 51.3%, respectively, both of which were lower than the proposed boundary average nucleotide identity value (95 to 96%) and digital DNA-DNA hybridization value (70%) for distinguishing species (51). These results demonstrated that Bacillus sp. TL12 is a novel species of the genus Bacillus.

The *bac* gene cluster consists of eight genes, encoding four highly homologous precursor peptides (*bacA1*, *bacA2*, *bacA3*, and *bacA4*), two putative membrane proteins with bPH2 domains (*bacM1* and *bacM2*), a HlyD family secretion protein (*bacT*), and a protein with an ATP-binding conserved domain (*bacE*) (Fig. 2C). The amino acid sequence identities of the four precursor peptides BacA1, BacA2, BacA3, and BacA4 ranged from 85.42 to 97.92%, with those of BacA1, BacA3, and BacA4 being particularly high at 93.75 to 97.92%. The closest reported bacteriocin precursor peptide of the four precursor peptides BacA1, BacA2, BacA3, and BacA4 is LnqZ (the precursor peptide of a leaderless bacteriocin, lacticin Z) (30), and their amino acid sequence identities are 45% (23 of 51), 47% (24 of 51), 45% (23 of 51), and 45% (23 of 51), respectively. In summary, Bacillus sp. TL12 was considered a novel bacteriocin-producing strain and was selected for further analysis.

### Purification of antimicrobial substances of Bacillus sp. TL12.

Bacillus sp. TL12 was activated overnight in LB medium, and the culture (1 mL) was transferred into LB medium (100 mL) and incubated at 30°C for 10 h (OD_600_ = 4.0) with shaking at 200 rpm. The supernatant (5 liters) was collected by centrifugation at 10,000 × *g* for 10 min and then incubated with 500 g Amberlite XAD-7 resin (Sigma-Aldrich, St. Louis, MO, USA) at 4°C for 24 h. The resin was washed sequentially with 3 liters double-distilled water (ddH_2_O) and 1.5 liters 30% (vol/vol) ethanol, and the adsorbed antimicrobial agents were eluted by 1 liter 80% (vol/vol) ethanol (pH 2.0). The eluate was condensed using a rotary vacuum evaporator at 45°C and freeze-dried with a vacuum freeze dryer. The obtained powder was dissolved in 5 mL ddH_2_O and centrifuged at 10,000 × *g* for 10 min. The obtained supernatant was referred to as the antimicrobial CE and was further purified by the reverse-phase high-performance liquid chromatography (Dionex Ultimate 3000 system). The mobile phase was Milli-Q water (0.1% trifluoroacetic acid) and acetonitrile. After 50 μL CE was loaded onto the C4 column (Vydamas VD0128), elution was performed with a linear gradient of 10 to 90% acetonitrile within 60 min at a 1 mL/min flow rate. The absorbance of the eluent was monitored at 220 nm. Subsequently, the antimicrobial activity of each of the fractions was tested against the indicator strain MRSA ATCC 43300 by the agar well diffusion method as described earlier. If two or more fractions had antimicrobial activities, their antimicrobial activity after mixing them was also tested to detect their synergistic antimicrobial activity. Each fraction containing the antimicrobial substance was manually collected 100 times and lyophilized into a powder. The acquired powder was dissolved in ddH_2_O and repeatedly purified with the above HPLC procedure five times to acquire the high purity of antimicrobial substance (>99%).

### Identification of antimicrobial substances of Bacillus sp. TL12.

Amino acid sequencing of antimicrobial substance of Bacillus sp. TL12 was performed by the Edman degradation method using a PPSQ-33A protein sequencer (Shimadzu, Japan). The exact mass of antimicrobial substances was performed on the Agilent 6540 ultrahigh definition accurate-mass quadrupole time-of-flight LC/MS system. MS conditions were adjusted to the following conditions: capillary temperature, 350°C; nebulizer pressure, 35 lb/in^2^; drying gas, 9 liters/min; and source voltage, 3.5 kV. The tandem mass spectrometry (MS/MS) spectrum analysis of antimicrobial substance was applied for fragmentation analysis of peptides, and the target ion was fragmented by adding a voltage of between 30 and 50 V.

### Determination of MIC and MBC of bacin A2.

The MICs of bacin A2 against indicator strains (Table 1) were measured using the microdilution method as described previously (29). Each indicator strain was grown overnight in the corresponding medium, and the culture was diluted to 1.0 × 10^6^ CFU/mL. Aliquots of 50 μL of the bacterial suspension and an equal volume 50 μL of bacin A2 solution were added to a 96-well microtiter plate and incubated at appropriate temperature for 18 h. We used the corresponding culture medium to dissolve and dilute bacin A2 according to different indicator bacteria (Table 1). For example, to test the MIC of bacin A2 against L. monocytogenes, bacin A2 was dissolved and diluted with TSB-YE medium. The final concentrations of bacin A2 were 128, 64, 32, 16, 8, 4, 2, 1, and 0.5 μM. The MIC was assessed as the minimum concentration that completely inhibited the growth of indicator strains. The MBC was determined as the lowest concentration at which no growth is observed after streaking the culture on corresponding solid medium.

### Biofilm susceptibility assay.

The effect of bacin A2 on S. aureus biofilm formation was examined using the microdilution method as described previously with minor modifications (52). Briefly, overnight cultures of S. aureus ATCC 6538 and MRSA ATCC 43300 grown in TSB were diluted to 1 × 10^6^ CFU/mL. A 100-μL aliquot and 100-μL 2-fold serial dilutions of bacin A2 with the final concentration ranging from 1/4× MIC to 2× MIC were added to a 96-well microtiter plate. Bacin A2 was dissolved and diluted with TSB medium. Nisin A (5 μM) and TSB medium were used as positive and negative controls, respectively. Nisin A was purified from commercial nisin A product (>900 IU/mg) (Cayman Chemical, Ann Arbor, MI, USA) as described previously (53). Following incubation at 37°C for 24 h, the bacterial growth (OD_600_) was assessed using a microplate reader. The biofilm assay was performed by discarding the culture supernatants, washing twice with phosphate-buffered saline (PBS), fixing with methanol for 20 min, and staining with 0.1% CV for 30 min. Subsequently, the CV was removed, and the wells were washed with water. Biofilm formation was quantified by the addition of 200 μL of 95% ethanol to each CV-stained biofilm cells, and the absorbance of solution at 570 nm was measured using a microplate reader.

To assess the effect of bacin on 1-day-old biofilm, 200 μL of 5 × 10^5^ CFU/mL of S. aureus ATCC 6538 and MRSA ATCC 43300 were respectively incubated into the wells of microtiter plates. After incubation at 37°C for 24 h, the culture supernatant from each well was removed, and the wells were washed twice with PBS. Bacin A2 (200 μL of various MICs) dissolved in TSB was added and incubated with the formed biofilm. After incubation at 37°C for 24 h, the supernatant in the wells was removed and washed once with PBS, and the bacin A2-treated biofilm was evaluated for viability using XTT assay as described previously (54). A 200 μL volume of XTT-menadione solution containing 0.50 mg/mL of XTT and 25 μM menadione were added to each well. The plates were incubated for 3 h at 37°C in the dark. The supernatant (100 μL) was transferred to the wells of a new 96-well flat-bottom plate, and the absorbance at 490 nm was measured. The viable count method (CFU) was also used to test the viability of S. aureus cells. Briefly, the biofilms were prepared as described earlier, scratched off, and suspended in 200 μL of sterile water. The suspension was diluted under a gradient and plated onto TSB plates. The viable count was measured after incubation at 37°C for 24 h. Nisin A (50 μM) and TSB medium were used as positive and negative controls, respectively.

### Mode of action of bacin A2.

**(i) Time-dependent killing of MRSA by bacin A2.** An overnight culture of MRSA ATCC 43300 was diluted with NB medium to an OD_600_ of 0.4 and incubated with bacin A2 at final concentrations of 0×, 1/2×, 1×, 2×, 4×, and 8× MIC at 37°C and 200 rpm. Bacin A2 was dissolved and diluted with NB medium. The absorbance of the culture at 600 nm was measured hourly to monitor the cell growth. NB medium was used as the negative control. After incubation for 3 h, each culture was photographed.

**(ii) Potassium efflux.** MRSA ATCC 43300 was cultured overnight, harvested using centrifugation method (10,000 × *g* for 10 min), washed, and resuspended in normal saline to an OD_600_ of 0.6. Subsequently, 1-mL aliquots were incubated with 2× and 4× MIC of bacin A2 at 30°C, and the total potassium ions (K^+^) in the supernatant were measured hourly using an HI96750 potassium photometer. Bacin A2 was dissolved and diluted with normal saline. Normal saline was used as the negative control.

**(iii) SEM.** Bacin A2 was dissolved and diluted with NB medium and was incubated with MRSA ATCC 43300 (OD_600_ = 0.4) to final concentrations of 2× and 4× MIC at 37°C for 3 h with gentle shaking. NB medium was used as the negative control. The cells were corrected by centrifugation (6,000 × *g* for 10 min), washed three times with PBS (0.1 mol/liters, pH 7.2), and fixed with 2.5% glutaraldehyde for 12 h. The cells were dehydrated using gradient alcohol solutions (10 to 100%) for 20 min each, treated with isoamyl acetate for 20 min, dried by a vacuum freeze dryer for 12 h, and sputter-coated with gold. The images were observed and acquired with SEM (Zeiss Sigma 300, Carl Zeiss NTS, Germany).

**(iv) Analysis of hemolytic activity.** Defibrillated sheep blood was centrifuged at 1,000 × *g* for 5 min, and the acquired blood cells were washed three times with PBS. The cells were resuspended with PBS and added to centrifugation tubes. Bacin A2 was dissolved in PBS and added to the tubes at final concentrations of 1, 16, and 64 μM. After incubation for 2 h at 37°C, the cells were centrifuged at 1,000 × *g* for 5 min. The supernatant was added to a 96-well microtiter plate, and the absorbance of each well was measured at 540-nm wavelength. PBS was taken as the negative control (0% hemolysis), and 1% Triton X-100 was taken as the positive control (100% hemolysis).

**(v) Analysis of cytotoxicity.** The cell counting kit 8 (CCK-8) assay was used to determine the cytotoxicity of bacin A2. Exponentially growing NIH/3T3 mouse embryonic fibroblast cells were added to a 96-well microtiter plate (5 × 10^3^ cells/well), and the plate was incubated at 37°C for 24 h. The medium was replaced with fresh medium (Dulbecco’s modified Eagle’s medium [DMEM] + 10% fetal bovine serum [FBS]) containing bacin A2 at final concentrations of 1, 16, and 64 μM. After 8 h of incubation at 37°C, CCK-8 solution (APExBIO, Houston, TX, USA) was added to the wells, and the plate was incubated at 37°C for 3 h. Finally, the absorbance of each well was measured at 450-nm wavelength. PBS was taken as the negative control, and paclitaxel (10 μg/mL) was taken as the positive control.

### Statistical analysis.

All experiments were performed in triplicate, and the data were statistically analyzed using SPSS 26.0. All data are presented as the means ± standard deviation. Significant differences (*P* values) between pairwise comparison with the control group were analyzed by Student’s *t* test, and *P ≤ *0.05 was considered statistically significant. Asterisk ratings indicate statistically significant differences: *, *P ≤ *0.05; **, *P ≤ *0.01; ***, *P ≤ *0.001; and ****, *P ≤ *0.0001.

### Data availability.

The 16S rRNA and genome sequence of Bacillus sp. TL12 were submitted to GenBank with the accession numbers OL774512.1 and JAJJBY000000000.1, respectively. The nucleotide sequences of the *bac* gene cluster were submitted to GenBank under the accession number OL456173.1.
