# Supplementary material for: Four Novel Leaderless Bacteriocins, Bacin A1, A2, A3, and A4 Exhibit Potent Antimicrobial and Antibiofilm Activities against Methicillin-Resistant Staphylococcus aureus
Source: Microbiol Spectr. 2022 Aug 24;10(5):e00945-22. doi: 10.1128/spectrum.00945-22 (PMC9602277; doi:10.1128/spectrum.00945-22)
Supplement: Supplemental file 1 — Table S1 and Fig. S1. Download spectrum.00945-22-s0001.pdf, PDF file, 0.7 MB [file spectrum.00945-22-s0001.pdf]

Four Novel Leaderless Bacteriocins, Bacin A1, A2, A3, and A4 Exhibit Potent Antimicrobial and Anti-biofilm Activities Against Methicillin-resistant *Staphylococcus aureus*

Shu Liu[a,*], Shulin Deng[a,*], Hualin Liu[b], Liang Tang[a], Mengqi Wang, Bingyue Xin[a, #], Feng Li[a, #]

[a]Anhui Province Key Laboratory of Pollutant Sensitive Materials and Environmental Remediation, College of Life Sciences, Huaibei Normal University, Huaibei, Anhui Province 235000, China

[b]School of Marine Sciences, Sun Yat-sen University, Zhuhai, Guangdong Province, 519000, China

[*]S.L. and S.D. contributed equally to this work. Author order was determined by their seniority and initial contribution.

[#]Corresponding authors:

Bingyue Xin, PhD E-mail address: xinbingyuex@163.com

Feng Li, PhD E-mail address: lifengmicro@163.com

Running title: Novel Bacteriocins with potent anti-MRSA activities

Keywords: Methicillin-resistant *Staphylococcus aureus* (MRSA), Leaderless Bacteriocin, Bacin

Table S1 Information about isolation source, species, prediction of novel bacteriocin gene cluster, and detection of anti-MRSA activity of the 1056 strains of *B. cereus* group used in this study

Isolation source	Species*	Number of strains	Number of predicted novel bacteriocin gene clusters	Number of strains that show anti-MRSA activity
Soil	*B. albus*	8	2	0
Soil	*B. bombysepticus*	5	1	0
Soil	*B. cereus*	351	198	21
Soil	*B. mycoides*	28	8	1
Soil	*B. thuringiensis*	505	251	27
Soil	*B. toyonensis*	67	13	2
Soil	*B. pseudomycoides*	23	3	0
Soil	*B. weihenstephanensis*	13	2	0
Soil	Unidentified species	56	24	5

*Species identification of these strains was performed using 16S rRNA gene sequence and comparative genome analyses (average nucleotide identity and digital DDH between genome sequences of the strains and type strain of a species).

Unidentified species—Through 16S rRNA gene sequence analysis, some strains, such as *Bacillus* sp. TL12, were determined for the *B. cereus* group strain. Comparative genome analysis showed they are novel species and need further phenotypic, physiological, biochemical, and molecular phylogenetic analyses.

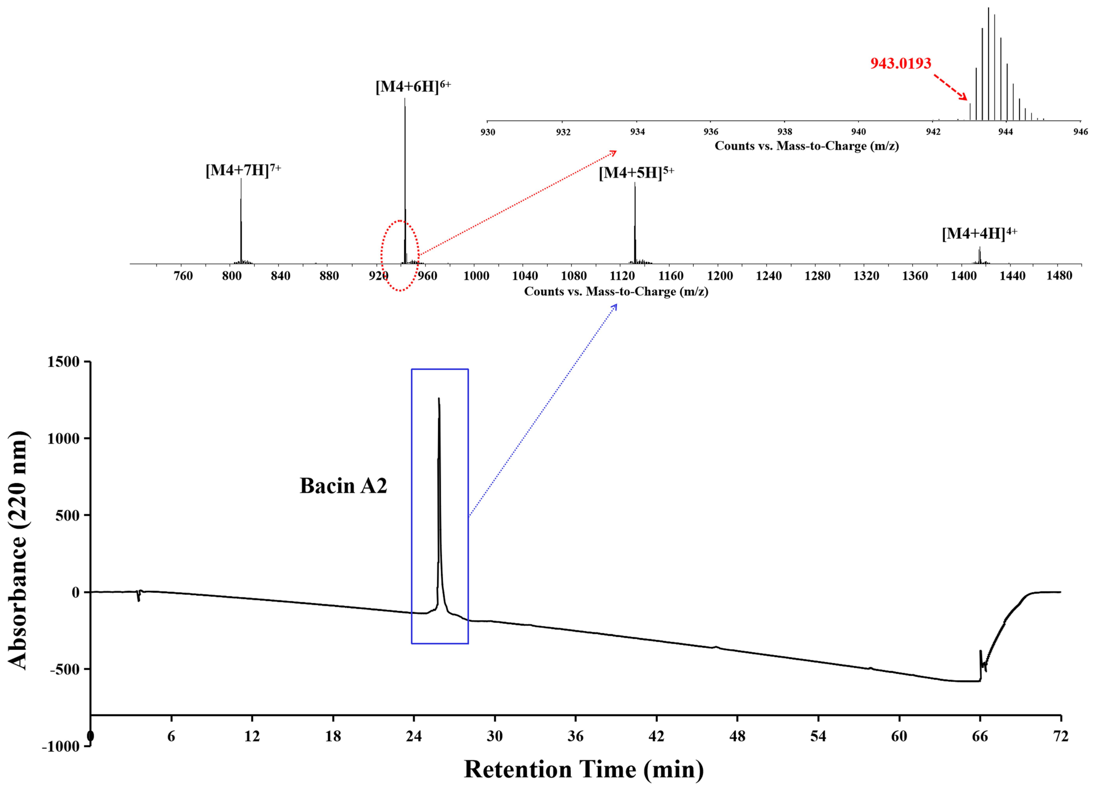

Fig. S1 HPLC-MS analysis of the purity of bacin A2, which was acquired by repeated HPLC collection and preparation. No contamination of other peptides (bacin A1, A3, and A4) was found in the bacin A2 sample by mass spectrometry analysis.
